# Domino Liver Transplantation from a Child with Propionic Acidemia to a Child with Idiopathic Fulminant Hepatic Failure

**DOI:** 10.1155/2018/1897495

**Published:** 2018-03-14

**Authors:** Marina Moguilevitch, Ellise Delphin

**Affiliations:** Department of Anesthesiology, Montefiore Medical Center, Albert Einstein College of Medicine, Bronx, NY 10463, USA

## Abstract

Domino liver transplant has emerged as a viable strategy to increase the number of grafts available for transplantation. In the domino transplant organs explanted from one patient are transplanted into another patient. The first successful domino liver transplant was performed in Portugal in 1995. Since then this innovative concept has been applied to several genetic or biochemical disorders that are treated by liver transplantation. An important consideration during this operation is that such livers can pose a risk of the de novo development of the disease in the recipient. That is why this surgical procedure requires careful planning, proper selection of the patients, and informed consent of both donor and recipient.

## 1. Introduction

With the ever-present shortage of liver grafts available for transplantation, new methods to expand the donor pool are being employed. In addition to split liver and living donors, the concept of “domino liver transplantation” (DLT) has been described as a way of utilizing an organ that would be discarded otherwise. Morphologically normal livers from donors with metabolic diseases can be used for select recipients. Usually, the metabolic disease of the donor should not produce symptoms in the DLT recipients for many years. In order to gather information on the DLT procedures an international register was created in 1999. By the end of 2015 a total 1112 domino transplantations were registered. They were reported from 63 different hospitals in 21 countries. There are no reports of use of the whole liver donor graft from the patient with propionic acidemia (PA).

## 2. Case Presentation

We would like to share our unique experience with the successful domino liver transplantation from a child with propionic acidemia to a young teenager with idiopathic fulminant hepatic failure. A 20 kg, O positive, 4-year-old female developed metabolic acidosis at birth and was diagnosed with PA by newborn screening. She required a protein restricted diet supplemented with Propimex formula and daily L-carnitine therapy for management. She had multiple propionic acidemia flares with hospital admissions for ammonia and urinary ketones elevation as well as dehydration secondary to poor oral intake. As a result of PA, she was developmentally delayed with impaired motor and verbal skills. Upon admission for possible transplantation, she was in her usual state of health with elevated ammonia level (73.9 *μ*mol/L) and urinary ketones (6.8 *μ*mol/L). The rest of the laboratory values including all liver function tests and coagulation studies were within normal limits.

When a cadaveric liver became available for this child there was a 14-year-old, blood type A positive, 50 kg male in the pediatric intensive care unit (PICU) with fulminant hepatic failure secondary to hepatitis of unknown etiology. Upon admission he had complaints of nausea and vomiting and on exam had scleral icterus. His admission laboratory values were notable for total bilirubin of 314.5 *μ*mol/L, direct bilirubin 217 *μ*mol/L, alkaline phosphatase 5.7 ukat/L, alanine aminotransferase 52.4 ukat/L, aspartate aminotransferase 64.4 ukat/L, international normalized ratio (INR) 2.1, and prothrombin time (PT) 32.3 seconds.

His course rapidly progressed to grade IV encephalopathy and fulminant hepatic failure necessitating intubation. His liver function deteriorated significantly with total bilirubin increase to 405 *μ*mol/L, ammonia 10.6 *μ*mol/L, INR 3.8, and PT 38 seconds. CT of the brain did not show any signs of edema. He was listed as a status 1 and an urgent liver transplant would be his only life-saving opportunity. Prior to the time of his listing a cadaveric liver graft offer had already been given to the child with propionic acidemia and no other suitable grafts were available. The team was concerned about rapidly deteriorating neurologic status of the patient. Temporizing measures of blood purification known to improve coagulation status but not proven to guarantee reversal of encephalopathy were discussed with the family. After waiting for about 24 hours parents were presented with the option of using the liver from the patient with propionic acidemia. The family had full understanding that a missing mitochondrial enzyme is also present in other organs of the body but the liver remains primary source of it. A careful review of the risks for a liver transplantation in critically ill patient as well as lack of data associated with transplanting the liver from the patient with propionic acidemia was conducted with the family. The family expressed full understanding and gave consent for the surgery. The family of the child with propionic acidemia was also approached with the permission to use the explanted liver. After obtaining informed consent for the surgery from both families domino liver transplant was performed. Donor liver was procured using classic technique. Venous outflow reconstruction was accomplished by end-to-side cavocaval anastomosis. To overcome a common problem of the short venous cuffs a bridge venoplasties technique between donor and recipient hepatic veins was utilized.

During the postoperative period the recipient with the transplanted propionic acidemia liver had received careful monitoring of his metabolic profile on monthly schedule, particularly urinary ketones and serum ammonia levels. His protein intake was initially restricted but ultimately liberalized and he was discharged to a rehabilitation facility tolerating a regular diet. His outpatient follow-up has been uneventful for two years with recommended checkup every 2 to 3 months. The family was presented with an option of possible retransplantation in the future in case of the development of propionic acidemia. Our 4-year-old patient with PA had one minor complication, an increase in liver function tests approximately 3 weeks after transplantation, causing concern about rejection. Liver biopsy showed scattered inflammation with neutrophils and lymphocytes consistent with infection rather than rejection, which eventually resolved. The patient was discharged home and is doing well.

## 3. Discussion

In 1995 domino liver transplantation emerged as a viable strategy to relieve the organ shortage. The first reported case came from Portugal where the liver from a patient with familial amyloidotic polyneuropathy (FAP) was used for non-FAP patient [1]. The domino approach can be considered for patients with some genetic or biochemical disorders that today are treated by liver transplantation. With the increasing need for organs, livers explanted from patients with primary hyperoxaluria (PH), acute intermittent porphyria (AIP), maple syrup urine disease (MSUD), and homozygous familial hypercholesterolemia (HFHC) are being used for DLT. The outcomes of these transplantations are variable: they are better in cases of MSUD and HFHC and are not as favorable in cases of PH and AIP. One of the latest developments in this area is the use of the grafts in neonates as a bridging therapy; this allows babies to grow until they can receive a size-compatible liver. The main risk of these surgeries includes de novo development of the genetic disorders in the recipients. Other important aspects to consider are the donors' survival as well as a need to keep mortality and morbidity of the recipients to a minimum [2].

This procedure raises very unique ethical issues. The most important one is informed consent of both donor and recipient with emphasis on the possibility of the recipient developing a metabolic disorder in the future. A hepatectomy procedure on a domino donor should meet the demands of the suitable graft and should not expose a donor to additional surgical risks. With the growing experience in DLT the main indication for the recipients remains hepatocellular carcinoma (HCC). Other possible indications listed in Domino Liver Transplant Registry are metastatic hepatic malignancy, cirrhosis secondary to hepatitis B and hepatitis C, alcoholic cirrhosis, and retransplantation.

To date, there have been no published case reports regarding the use of whole liver from patients with propionic academia (PA) for a DLT. PA is an autosomal recessive disease classified as a branched chain organic aminoacidemia. It is caused by a reduction in the activity of enzyme propionyl coenzyme A carboxylase (PCC). This occurs during the *β*-oxidation of odd chain fatty acids, catabolism of methionine, leucine, isoleucine, threonine, and valine, or metabolism of the cholesterol side chain. This altered metabolism leads to metabolic acidosis and hyperammonemia. There are two clinical phenotypes: (1) neonatal, the most common, and (2) late onset, less common. Clinical manifestations of the disease are poor feeding, vomiting, lethargy, and developmental delay leading to rapid neurologic deterioration, failure to thrive, and cardiomyopathy. PCC is also being produced in extrahepatic locations, mainly gut and central nervous system, which makes these patients not ideal candidates for liver transplantation as a potential cure for the disease [3]. The use of liver transplantation for patients with PA has been described in several case study series as one of the potential treatment modalities in reducing the risks of metabolic decompensation, neurocognitive dysfunction, and quality of life [4, 5]. The domino reuse of livers affected by propionic acidemia should provide hepatic rescue therapy for the recipient and theoretically avoid metabolic crisis because of enzyme production in extrahepatic sites [6]. According to the literature, DLT using grafts affected by a metabolic disorder could be an option for recipients who fulfill at least one of the following criteria: presence of normal systemic enzyme activity that can compensate for the genetic defect transmitted by the graft; an expected interval before the onset of the acquired metabolic disease that is longer than expected survival without liver transplantation; a bridge therapy while waiting for the healthy graft [7].

Postoperative management of the recipient of the PA liver involves initially restricting protein intake, as one would do in a patient with PA. Close follow-up of metabolic profile, particularly urinary ketones and serum ammonia, is very important. Liver transplantation plays a major role in survival as well as quality of life improvement of patients suffering from propionic acidemia. The ideal timing of transplantation for patients with this metabolic disorder is still unknown. There is ongoing debate about prioritization of these candidates on transplant list. We still do not know what quality of organ is acceptable for these non-life-saving transplantations. At this point in time it behooves us to report every new experience in transplantation for patients with metabolic disorders and especially the successful use of explanted liver from these patients as donors. This will expand our knowledge in the relatively new area of domino liver transplantation and allow this novel strategy to be used to increase the number of liver grafts available.

We report this as one of the first DLT procedures utilizing a liver from a patient with PA that resulted in salvage and recovery of a patient with fulminant hepatic failure.
